# Genomic Organization of Human Transcription Initiation Complexes

**DOI:** 10.1371/journal.pone.0149339

**Published:** 2016-02-11

**Authors:** B. Franklin Pugh, Bryan J. Venters

**Affiliations:** Center for Eukaryotic Gene Regulation, Department of Biochemistry and Molecular Biology, The Pennsylvania State University, University Park, Pennsylvania, United States of America; Università degli Studi di Milano, ITALY

## Abstract

A repertoire of transcription initiation factors engage the core promoter of mRNA genes to recruit RNA polymerase (Pol) II to initiate transcription, yet their precise spatial organization remains unclear. Using ChIP-exo, here we detail the interactions and genomic organization of initiation factors TBP, TFIIB, and Pol II at mRNA genes and within CpG islands. We find that when Pol II moves into a transcriptionally paused state, TBP/TFIIB remain at the promoter. We show that TBP and TFIIB bound to the core promoter at two separate, resolvable locations that coincided with sites of divergent transcription initiation. We also examine the precise binding of TBP at Pol III transcribed tRNA genes. We find that TBP crosslinked to tRNA genes in a similar manner as at Pol II transcribed genes. This comprehensive and high resolution genome-wide detection of the initiation machinery produces a consolidated view of transcription initiation events humans at Pol II coding and Pol III transcribed tRNA genes.

## Introduction

The classic paradigm for assembling the minimal core transcription machinery at mRNA promoters starts with the recruitment of the TATA binding protein (TBP). Next is the docking of TFIIB, which straddles and locks onto TBP. Together with TFIIF, TFIIB then engages Pol II in its active site to help set the start site of transcription (TSS) [1, 2]. The recruitment of the transcription machinery has long been thought to be an important rate-limiting step in gene expression [3]. Concepts in transcription initiation by all three RNA polymerases (I, II, and III) have been guided by this basic theme [4].

For RNA polymerase II, in multi-cellular eukaryotes, some of general transcription factors may be largely pre-assembled at promoters. There, Pol II is in a transcriptionally engaged but paused state, approximately 30–50 bp downstream from the TSS [5–7]. Further complicating the classic paradigm of transcription initiation of mRNA genes is the coupling of antisense transcription upstream of the core promoter [8]. These divergent TSSs are spaced roughly 250 bp apart with some variance, and driven by separate initiation complexes [9]. However, the precise genomic organization of human transcription complexes within this context remains unclear. Conventional genomic factor mapping approaches, such as ChIP-seq, are not of sufficiently high resolution to address this issue.

Most vertebrate promoters are found within stretches DNA with high density of CG dinucleotides, called CpG islands, that can be reversibly methylated [10]. Methylation of CpG islands is associated with transcriptional silencing, whereas unmethylated or hypomethylated CpG islands are thought to contribute to creating a permissive chromatin state for transcriptional activation by destabilizing nucleosomes [11]. The prevalence of CpG islands at promoters and their influence on transcription raises the question of how the transcription initiation machinery is organized within this genomic context.

In contrast, transcription initiation by RNA polymerase III at tRNA genes involves TFIIIC recognition of specific sequences downstream of the TSS, then recruits TFIIIB to a region immediately upstream of the TSS that lacks apparent sequence specificity [12, 13]. Pol III then binds to form a pre-initiation complex. TFIIIB contains TBP (and BRF, a factor related to TFIIB) and thus it has been enigmatic as to how TBP in TFIIIB engages the upstream region.

In 2013, we published a manuscript detailing the organization of the TBP, TFIIB, and Pol II (PIC) components of the transcription machinery across the human genome in K562 cells and other transformed cell lines using the ChIP-exo genome-wide assay [14]. That paper was retracted in 2014 due to statistical errors concerning the specificity of DNA sequence elements associated with the identified PICs. Here we report those parts of the study that we deem to remain valid. This includes a characterization of the structural organization of TBP, TFIIB, and Pol II around coding genes and TBP at tRNA genes.

## Results

### Pre-initiation complex occupancy (PIC) at mRNA genes

**Fig 1:**
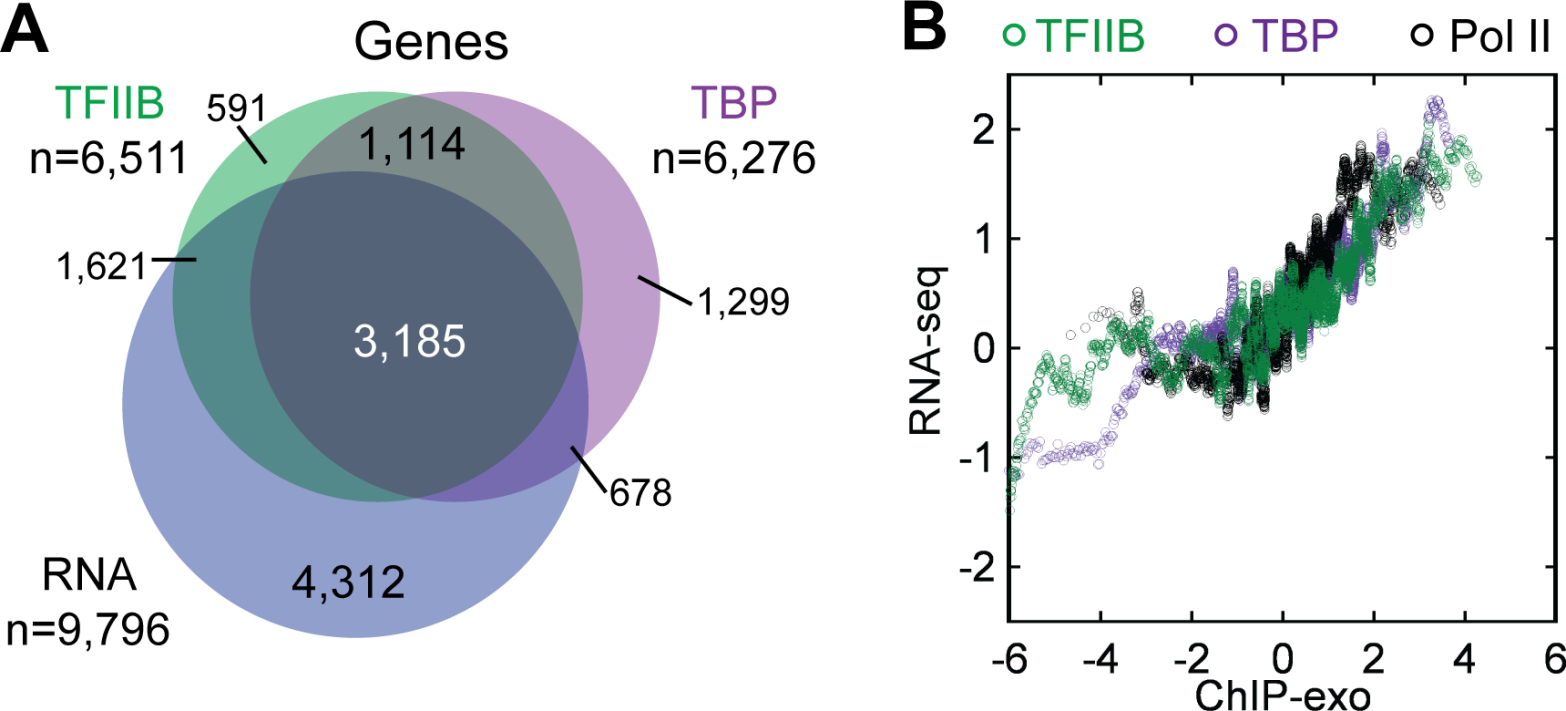
Overlap and trend of ChIP-exo data at mRNA genes. (A) Venn overlap among mRNA genes having TBP or TFIIB locations (<500 bp from its TSS) and genes with measured polyadenylated mRNA levels detected by RNA-seq [39]. Data thresholding may contribute to nonoverlapping sets. (B) Moving average (100-gene) of mRNA levels versus TFIIB/TBP/Pol II occupancy levels on a median-centered log_2_ scale.

To obtain a detailed assessment of pre- and post-initiation complexes we conducted ChIP-exo on TBP, TFIIB, and Pol II in the human erythroleukemia cell line (K562). We focused on TBP and TFIIB to assess PIC formation because in yeast these proteins were the most detail-rich, whereas other initiation factors displayed essentially similar ChIP-exo patterns [15]. To assess post-initiation transcription complexes and the extent to which genes display promoter-proximal pausing, we ChIP’d the largest Pol II subunit (POLR2A). 8,364 TFIIB ChIP-exo peak-pairs (Table A in S1 File) were found within 500 bp of an mRNA TSS, which corresponds to ~50% of all annotated protein-coding K562-expressed genes (Fig 1A). Seemingly expressed genes that lacked a TBP/TFIIB location may have arisen from multiple sources including rare but stable mRNAs, detection noise, and antisense transcription arising from a more distal promoter. TBP/TFIIB/Pol II occupancy and mRNA levels displayed a similar trend (Fig 1B), but were weakly correlated (S1 Fig), possibly due to differences in RNA stability.

### Divergent mRNA transcripts arise from distinct initiation complexes

**Fig 2:**
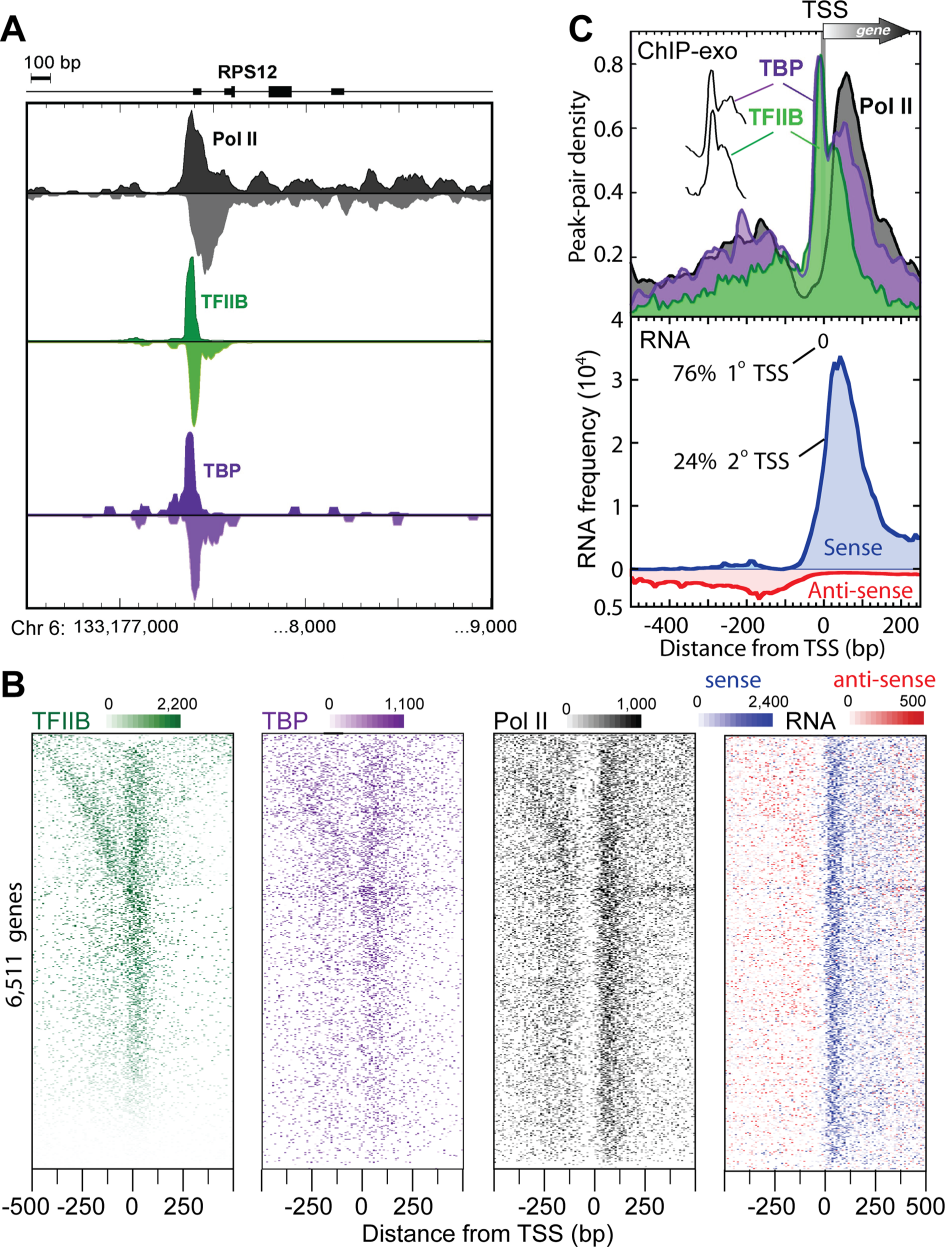
Transcription machinery organization at human mRNA promoters. (A) Smoothed distribution of strand-separated ChIP-exo tag 5′ ends at the *RPS12* gene. (B) ChIP-exo peak-pair or RNA distribution at RefSeq genes (rows). Rows are linked, and sorted by distance between adjacent TFIIB peak-pairs. (C) Upper panel: Averaged ChIP-exo patterns around the closest (1**°**) RefSeq TSS. Peak-pair tags were aligned to the TSS gene-by-gene, binned in non-overlapping 10 bp intervals relative to the TSS, and then the average peak-pair density value across all TFIIB-occupied (n = 6,511) genes was plotted as a percent of the total. The “spikes” of TBP and TFIIB are indiscernible (vertically offset in inset). Lower panel: Distribution of 2**°** polyadenylated RNA [39], with traces separated by sense (blue) and antisense (red, inverted trace) orientations relative to the corresponding mRNA TSS.

To gain detailed insight into the structural organization of human promoter initiation complexes, we focused on the 8,364 K562 TFIIB locations near the TSS of 6,511 coding RNAs as defined by RefSeq [16]. Fig 2A provides one example of the raw tag distribution concentrated ~25 bp upstream of the *RPS12* ribosomal protein gene TSS. When individual genes were examined (Fig 2B), or averaged (meta analysis) across all 6,511 genes (Fig 2C), two regions of high TFIIB/TBP/Pol II occupancy were observed. The major right-ward peaks corresponded to primary promoter transcription initiated complexes (Fig 2C, upper panel). Those in the left-ward direction matched divergent TSSs [8, 17–19], although the resulting RNA was less abundant than expected from TFIIB/TBP/Pol II occupancy levels (Fig 2C, lower vs upper panel; Note that 2° TSS represents only 24% of the total TSS signal). This may result from RNA instability, as seen in yeast [20–22]. The clear spatial separation of complexes indicates that divergent transcripts arise from distinct initiation complexes.

### TBP/TFIIB separation from paused Pol II in CpG islands

**Fig 3:**
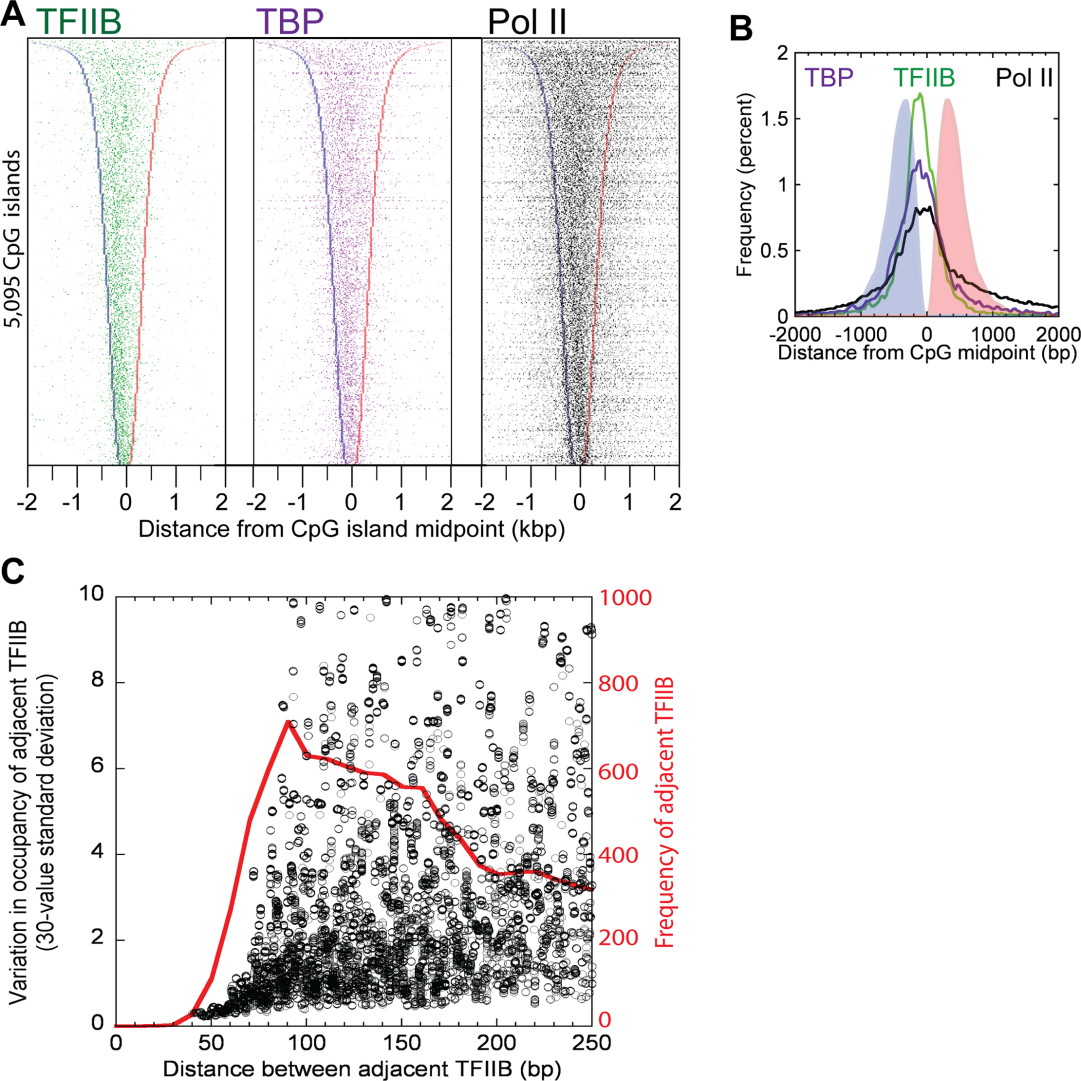
Distribution of the TFIIB/TBP/Pol II in CpG islands that overlap mRNA TSSs. (A) Peak-pair distribution for TFIIB, TBP and Pol II at the 5,095 CpG islands that overlap with the 6,511 mRNA TSSs from Fig 1B (78% overlap), and with the direction of transcription to the right. Rows are linked, and sorted by CpG island length. CpG island left and right borders are indicated by blue and red bars, respectively. (B) Shown is the averaged data from panel A. (C) TFIIB locations were sorted by location, and inter-TFIIB distances calculated (red trace). Data were then sorted by distance, and the standard deviation of TFIIB occupancy was calculated on a sliding window of 30 values. Peak calling parameters preclude detection of two separate TFIIB locations <~40 bp apart. Those that were 40–70 bp apart were correlated, whereas those >~70 bp apart were uncorrelated.

CpG islands overlapped with nearly 80% (5,095) of the 6,511 mRNA gene promoters where we detected TFIIB ChIP-exo crosslinking. We found that on average 1.6 TFIIB complexes (8,254 locations within 5,095 CpG islands) were detected per CpG island, regardless of island length, with the center of the island being enriched ~100 bp downstream of the primary TSS (Fig 3A and 3B). Those complexes separated by >80 bp had uncorrelated occupancies (Fig 3C, black), which suggests that they are regulated independently. Those, <80 bp apart appeared to be regulated coordinately.

For the vast majority of transcription units, Pol II crosslinked 50 bp downstream of the primary TSS (Fig 2B and 2C), where it is expected to pause after initiating transcription [7]. Pol II was most depleted 20–60 bp upstream of the consensus TSS, indicating that on average it likely spends less time there than at the paused sites, in proliferating K562 cells. This suggests that in most (but not necessarily all) cases, once Pol II is recruited it, rapidly clears the promoter and assumes a paused-state ~30–50 bp downstream of the TSS, consistent with the observation that Pol II pause release is a rate-limiting step in transcription [23, 24].

The crosslinking pattern of human TFIIB was of particular interest since TFIIB in budding yeast crosslinks broadly across the relatively stable single-stranded DNA region within the Pol II active site at core promoters [15], in accord with crystallographic models of “open” complexes [25]. However, with the majority of human Pol II residing at pause sites just downstream of the core promoter, and RNA rather than TFIIB filling the Pol II active site region, the human TFIIB crosslinking patterns may not resemble yeast. Remarkably, the major crosslinking point for human TFIIB was ~20 bp upstream of the TSS, rather than spread across the TSS as in yeast. This location is precisely where TFIIB contacts DNA immediately downstream of TBP bound to the TATA box. Additional lower intensity crosslinking was observed near the TSS (Fig 2A and 2C, upper panel), which would be consistent with low levels of TFIIB interactions with Pol II, either in the active site in a potential pre-initiation complex or elsewhere on a paused Pol II.

We identified 150,753 putative low-threshold (>4 tag counts) TFIIB locations in K562 cells that were far (>500 bp) from the TSS of a protein-coding gene. Of these, 21,160 were also identified in the TBP dataset (within 20bp), 9,192 in the Pol II dataset (within 150 bp), and 2,353 in a no-antibody control (within 150 bp, data not shown). About 57% and 5% of these TBP/TFIIB (11,192 of 19,952) and TBP/TFIIB/Pol II (47 of 1,033) co-occupied locations (and not in the no-antibody control), respectively, resided in ENCODE-annotated [26] heterochromatic regions of the genome. The cohort of 11,192 TBP/TFIIB promoter distal locations appear to be mostly (57%) isolated heterochromatic complexes whose physiological significance remains to be determined, although 36% of these locations did reside in ENCODE-annotated promoter/enhancer/transcription regions of the genome. In contrast, the vast majority (90%) TBP/TFIIB/Pol II co-occupied locations (n = 1,033) resided in ENCODE-annotated promoter/enhancer/transcription regions of the genome.

### TFIIB locations across cancer cell lines

**Fig 4:**
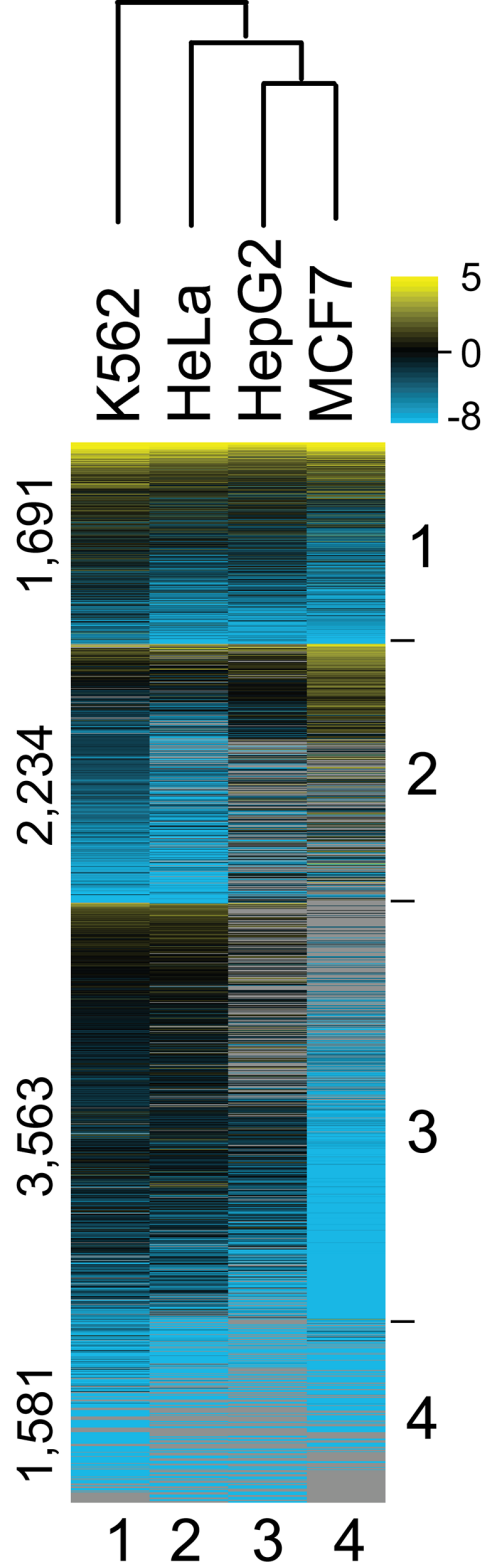
Promoter complexes across cancer cell lines. Occupancy levels for TFIIB linked to RefSeq TSSs (within 500 bp) in the indicated cell type were normalized by column. The color scales represent the range of average-centered, log_2_ transformed values within each respective column. Detection in all four cell types define Group 1. Groups 2–4 were parsed by k-means clustering. Rows were sorted within groups based on TFIIB occupancy averaged across the four cell types (yellow-black-cyan-gray, denote high, medium, low, and zero occupancy, respectively). The number of genes in each cluster group are indicated to the left.

To assess the extent to which TFIIB occupancy at coding genes differed across cancer cell lines, we conducted ChIP-exo mapping of TFIIB locations across four ENCODE cancer cell lines: HeLa S3, HepG2, and MCF7 in addition to K562 (cervical, liver, breast, and blood, respectively). We detected TFIIB at 9,069 mRNA genes in at least one cell line, and at 1,691 genes in all lines (group 1) (Fig 4 and Table B in S1 File). The remaining 7,378 genes were parsed by K-means clustering into three additional groups. For group 1, gene ontology analysis [27] revealed that these genes tend to be housekeeping genes, such as those involved in translation, chromatin assembly, and RNA splicing (*P* = 10^−55^, 10^−12^, and 10^−11^; respectively). As expected for housekeeping genes, these genes displayed similar levels of TFIIB occupancy across the four cancer cell lines tested. Groups 2 displayed some notable differences in occupancy, suggesting tissue-specific TFIIB promoter occupancy for some of the genes. In particular for group 2 genes, relative TFIIB occupancy was increased in HepG2/MCF7 and decreased in K562/HeLa. Group 2 was enriched with genes encoding transcription activators (*P* = 10^−6^), consistent with the frequent tissue-specific expression of TFs [28, 29]. Groups 3 and 4 comprised much of the lowly occupied (blue) genes across the four cancer cell lines, suggesting that these genes may be expressed at a basal level. For groups 3 and 4, gene ontology analysis showed an enrichment of RNA processing, catabolic, and cell cycle genes (*P* = 10^−15^, 10^−15^, and 10^−16^; respectively) that may not be as highly expressed as the translation (eg: ribosome protein subunits) and chromatin assembly (e.g., histones) found in group 1 that are among the most highly transcribed genes. MCF7and HepG2 TFIIB profiles were most distant from K562 on the dendrogram plot (Fig 4, above heatmap) suggesting that the patterns of TFIIB occupancy differed most between blood (K562) and breast/liver (HepG2/MCF7) tissues.

### TBP binds ~21 bp upstream of tRNA gene TSSs

**Fig 5:**
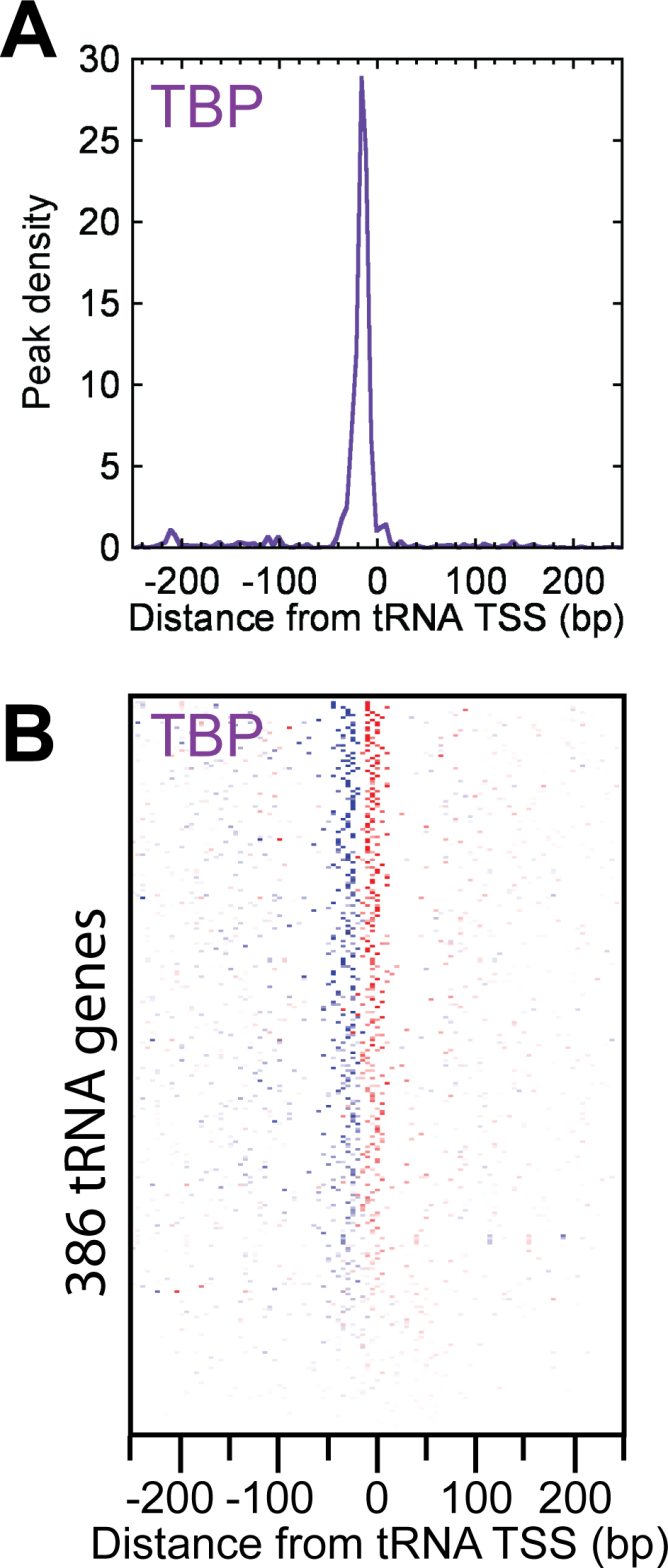
Organization of TBP at tRNA genes. (A) Average distribution of TBP peak-pairs around each tRNA TSS. (B) TBP peak density separated by forward and reverse strand orientation (blue and red colors, respectively) relative to each tRNA TSS.

Given that the initiation of Pol III genes requires a distinct set of complexes from Pol II genes, yet share TBP in common, we examined whether TBP crosslinked to tRNA genes in a similar manner as at Pol II transcribed genes. We aligned strand-separated exonuclease stop sites to the TSS of all 386 tRNA genes at which TBP was detected (Fig 5B). Remarkably, as evidenced by the peak-pair mode, TBP crosslinked ~21 bp upstream of 386 tRNA genes (Fig 5A**)**, which is in line with what was observed at Pol II promoters. Unlike at Pol II promoters, almost no crosslinking was observed downstream of the TSS, which may reflect a lack of pausing of Pol III (through which TBP might crosslink), which differs from Pol II. Similar to TBP crosslinking through TFIIB, TBP might predominantly crosslink through BRF. Indeed, the peaks of BRF and TBP crosslinking are coincident at Pol III genes in mice [30]. If true, then TBP in complex with a TFIIB family member might engage the core promoter similarly in Pol II and III systems.

## Discussion

### Consolidated genomic view of initiation

Genome-wide mapping of the general transcription machinery at near single-base resolution offers a clearer view of certain transcription initiation events from yeast to humans, Pol II to Pol III, and mRNA to tRNA. In general, a TFIIB/BRF family member is recruited to all coding or noncoding core promoters via a TBP family member in a spatially-constrained manner. As established elsewhere [24, 31–33], Pol II then scans downstream, where it encounters a TSS that allows for productive transcription. In metazoans, Pol II subsequently pauses 30–50 bp further downstream. In yeast, a nucleosome border may help set the start site of productive transcription [34]. Although core promoter regions are seemingly long (~40 bp in human) for sequence-specific binding, they do not appear to be enriched with well-defined sequence elements and so may have evolved to have inherently low specificity, presumably to keep basal transcription low and to maintain high dependence on transcriptional activators.

Divergent antisense transcription is a common feature of mammalian promoters [8, 18]. Whether these divergent transcription events arise from distinct PICs was unclear and unresolvable with the lower resolution of conventional ChIP-seq. We show that TBP and TFIIB bound to core promoter regions at two separate resolvable locations that coincided with sites of divergent transcription initiation. A recent study confirmed our original findings and interestingly extended them to suggest a unified architecture of bidirectional transcription initiation at promoters and enhancers [9].

Although the transcription of tRNA genes requires an almost entirely distinct set of machinery, TBP subunit nevertheless crosslinks at the same genomic position relative to TSSs of Pol II and Pol III transcribed tRNA genes. Therefore, TBP in complex with TFIIIB may engage the core promoter in Pol II and III systems in a fundamentally similar manner.

## Materials and Methods

### Cell Culture

Human chronic myelogenous leukemia cells (K562, ATCC) were maintained between 1x10^5^ – 1x10^6^ cells/milliliter in DMEM media supplemented with 10% bovine calf serum at 37°C with 5% CO_2_. Cells were washed and phosphate buffered saline (1x PBS, 8 mM Na_**2**_HPO_4_, 2 mM KH_2_PO_4_, 150 mM NaCl, and 2.7 mM KCl) before incubation with formaldehyde in a final concentration of 1% for 10 minutes. Cells were lysed (10 mM Tris pH 8, 10 mM NaCl, 0.5% NP40, and complete protease inhibitor cocktail (CPI, Roche), and then the nuclei lysed (50 mM Tris pH 8, 10 mM EDTA, 0.32% SDS, CPI). Purified chromatin was resuspended in IP dilution buffer (40 mM Tris pH 8.0, 7 mM EDTA, 56 mM NaCl, 0.4% Triton x-100, 0.2% SDS, and CPI) and sonicated with a Bioruptor (Diagenode) to obtain fragments with a size range between 100 and 500 bp.

### ChIP-exo and Antibodies

With the following modifications, ChIP-exo was carried out as previously described [35] with chromatin extracted from 10 million cells, ProteinG MagSepharose resin (GE Healthcare), and 3 ug of either TFIIB (Santa Cruz Biotech, sc-225), TBP (Santa Cruz Biotech, sc-204), or Pol II (Santa Cruz Biotech, sc-899, directed against the N-terminus of the Pol II large subunit encoded by POL2RA).

### Alignment to Genome, Peak Calling, and Data Access

Libraries were sequenced on an Illumina HiSeq sequencer. The entire length of the sequenced tags were aligned to the human hg18 reference genome using BWA [36] using default parameters. Raw sequencing data are available at NCBI Sequence Read Archive (SRA067908) and a sequencing statistics summary may be found in Table C in S1 File. The resulting sequence read distribution was used to identify peaks on the forward (W) and reverse (C) strand separately using the peak calling algorithm in GeneTrack (sigma = 20, exclusion zone = 40 bp) [37]. For strand-specific and strand-merged plots, sequencing tags were normalized to input. To obtain RPKM (Reads Per Kilobase per Million mapped reads) normalized counts, Input tags were binned relative to TSSs in the same manner as ChIP data, RPKM computed, and then for each corresponding bin the RPKM for each ChIP data set was divided through by the Input RPKM. Peaks were paired if they were 0–80 bp in the 3′ direction from each other and on opposite strands. Any peak-pair locations that were present in the ENCODE designated blacklist were removed from the analysis [38]. Since patterns described here were evident among individual biological replicates, and replicates were well correlated, we merged all tags from biological replicate data sets to make final peak-pair calls. TFIIB peak pairs in K562 cells were initially considered for preliminary analysis if they had a low threshold tag count of >4 in the merged datasets. 159,117 locations met these criteria. Subsequent analysis revealed that 8,364 TFIIB locations were near (within 500bp) a RefSeq TSS, while the remaining 150,753 TFIIB locations were TSS-distal (>500bp). Input tag distribution relative to 8,364 TFIIB locations is shown in S2 Fig.

NCBI-curated RefSeq TSSs (n = 26,987) [16] comprising 23,562 nonredundant gene TSSs genes were considered (Table D in S1 File). Assignment of TFIIB (8,364 peak-pairs) and TBP (7,642 peak-pairs) to the nearest RefSeq TSS required that they be within 500 bp of the TSS, yielding 6,511 nonredundant mRNA genes. Importantly, using a more stringent interval only marginally changed these numbers and did not alter our conclusions. If a gene had >1 TSS, then the TSS nearest to the bound location (peak-pair midpoint) was used as the primary TSS, and other nearby TSSs were considered secondary (Fig 1C, lower panel).

## Supporting Information

S1 Fig Correlation of ChIP-exo data at mRNA genes (related to main Fig 1). (A-C) Scatter plot and Pearson correlation fit of mRNA levels (RNA-seq) versus TFIIB/TBP/Pol II ChIP-exo occupancy levels, respectively, on a median-centered log_2_ scale. (D-F) Scatter plot and Pearson correlation fit of TFIIB/TBP/Pol II ChIP-exo occupancy levels versus each other on a median-centered log_2_ scale.
(TIF)

S2 Fig Input tag distribution relative to TFIIB locations (related to main Fig 2). Input sequence tags are plotted relative to the 8,364 promoter-bound TFIIB locations from main Fig 2B. as an averaged composite distribution (A) or as a density plot (B).
(TIF)

S1 File This file contains Tables A-D. Table A: TFIIB locations at protein coding genes, Table B: TFIIB occupancy across cancer cell lines, Table C: Sequencing statistics, and Table D: hg18 RefSeq Annotation.
(XLSX)

## References

[1] He Y, Fang J, Taatjes DJ, Nogales E. Structural visualization of key steps in human transcription initiation. Nature. 2013 Epub 2013/03/01. 10.1038/nature11991 . 23446344 PMC3612373

[2] Kostrewa D, Zeller ME, Armache KJ, Seizl M, Leike K, Thomm M, et al RNA polymerase II-TFIIB structure and mechanism of transcription initiation. Nature. 2009;462(7271):323–30. Epub 2009/10/13. 10.1038/nature08548 . 19820686

[3] Ptashne M, Gann A. Transcriptional activation by recruitment. Nature. 1997;386(6625):569–77. Epub 1997/04/10. 10.1038/386569a0 . 9121580

[4] Vannini A, Cramer P. Conservation between the RNA polymerase I, II, and III transcription initiation machineries. Molecular cell. 2012;45(4):439–46. Epub 2012/03/01. 10.1016/j.molcel.2012.01.023 . 22365827

[5] Gilmour DS, Lis JT. RNA polymerase II interacts with the promoter region of the noninduced hsp70 gene in Drosophila melanogaster cells. Mol Cell Biol. 1986;6(11):3984–9. . 3099167 10.1128/mcb.6.11.3984-3989.1986 PMC367162

[6] Guenther MG, Levine SS, Boyer LA, Jaenisch R, Young RA. A chromatin landmark and transcription initiation at most promoters in human cells. Cell. 2007;130(1):77–88. . 17632057 10.1016/j.cell.2007.05.042 PMC3200295

[7] Kwak H, Fuda NJ, Core LJ, Lis JT. Precise maps of RNA polymerase reveal how promoters direct initiation and pausing. Science. 2013;339(6122):950–3. Epub 2013/02/23. 10.1126/science.1229386 . 23430654 PMC3974810

[8] Core LJ, Lis JT. Transcription regulation through promoter-proximal pausing of RNA polymerase II. Science. 2008;319(5871):1791–2. Epub 2008/03/29. 10.1126/science.1150843 18369138 PMC2833332

[9] Core LJ, Martins AL, Danko CG, Waters CT, Siepel A, Lis JT. Analysis of nascent RNA identifies a unified architecture of initiation regions at mammalian promoters and enhancers. Nat Genet. 2014;46(12):1311–20. 10.1038/ng.3142 25383968 PMC4254663

[10] Saxonov S, Berg P, Brutlag DL. A genome-wide analysis of CpG dinucleotides in the human genome distinguishes two distinct classes of promoters. Proc Natl Acad Sci U S A. 2006;103(5):1412–7. 10.1073/pnas.0510310103 16432200 PMC1345710

[11] Zhang H, Zhu JK. Active DNA demethylation in plants and animals. Cold Spring Harb Symp Quant Biol. 2012;77:161–73. 10.1101/sqb.2012.77.014936 23197304 PMC3657592

[12] Geiduschek EP, Tocchini-Valentini GP. Transcription by RNA polymerase III. Annual review of biochemistry. 1988;57:873–914. Epub 1988/01/01. 10.1146/annurev.bi.57.070188.004301 . 3052292

[13] White RJ, Jackson SP. Mechanism of TATA-binding protein recruitment to a TATA-less class III promoter. Cell. 1992;71(6):1041–53. Epub 1992/12/11. . 1458535 10.1016/0092-8674(92)90398-v

[14] Venters BJ, Pugh BF. Genomic organization of human transcription initiation complexes. Nature. 2013;502(7469):53–8. 10.1038/nature12535 24048476 PMC4018585

[15] Rhee HS, Pugh BF. Genome-wide structure and organization of eukaryotic pre-initiation complexes. Nature. 2012;483(7389):295–301. Epub 2012/01/20. 10.1038/nature10799 22258509 PMC3306527

[16] Pruitt KD, Tatusova T, Maglott DR. NCBI reference sequences (RefSeq): a curated non-redundant sequence database of genomes, transcripts and proteins. Nucleic Acids Res. 2007;35(Database issue):D61–5. Epub 2006/11/30. 10.1093/nar/gkl842 17130148 PMC1716718

[17] He Y, Vogelstein B, Velculescu VE, Papadopoulos N, Kinzler KW. The antisense transcriptomes of human cells. Science. 2008;322(5909):1855–7. Epub 2008/12/06. 10.1126/science.1163853 19056939 PMC2824178

[18] Seila AC, Calabrese JM, Levine SS, Yeo GW, Rahl PB, Flynn RA, et al Divergent transcription from active promoters. Science. 2008;322(5909):1849–51. Epub 2008/12/06. 10.1126/science.1162253 19056940 PMC2692996

[19] Fenouil R, Cauchy P, Koch F, Descostes N, Cabeza JZ, Innocenti C, et al CpG islands and GC content dictate nucleosome depletion in a transcription-independent manner at mammalian promoters. Genome Res. 2012;22(12):2399–408. Epub 2012/10/27. 10.1101/gr.138776.112 23100115 PMC3514669

[20] Neil H, Malabat C, d'Aubenton-Carafa Y, Xu Z, Steinmetz LM, Jacquier A. Widespread bidirectional promoters are the major source of cryptic transcripts in yeast. Nature. 2009;457(7232):1038–42. 10.1038/nature07747 . 19169244

[21] Schulz D, Schwalb B, Kiesel A, Baejen C, Torkler P, Gagneur J, et al Transcriptome surveillance by selective termination of noncoding RNA synthesis. Cell. 2013;155(5):1075–87. 10.1016/j.cell.2013.10.024 . 24210918

[22] Xu Z, Wei W, Gagneur J, Perocchi F, Clauder-Munster S, Camblong J, et al Bidirectional promoters generate pervasive transcription in yeast. Nature. 2009;457(7232):1033–7. 10.1038/nature07728 19169243 PMC2766638

[23] Liu L, Xu Y, He M, Zhang M, Cui F, Lu L, et al Transcriptional pause release is a rate-limiting step for somatic cell reprogramming. Cell Stem Cell. 2014;15(5):574–88. 10.1016/j.stem.2014.09.018 . 25312495

[24] Min IM, Waterfall JJ, Core LJ, Munroe RJ, Schimenti J, Lis JT. Regulating RNA polymerase pausing and transcription elongation in embryonic stem cells. Genes Dev. 2011;25(7):742–54. 10.1101/gad.2005511 21460038 PMC3070936

[25] Sainsbury S, Niesser J, Cramer P. Structure and function of the initially transcribing RNA polymerase II-TFIIB complex. Nature. 2012 Epub 2012/11/16. 10.1038/nature11715 . 23151482

[26] Ernst J, Kheradpour P, Mikkelsen TS, Shoresh N, Ward LD, Epstein CB, et al Mapping and analysis of chromatin state dynamics in nine human cell types. Nature. 2011;473(7345):43–9. Epub 2011/03/29. 10.1038/nature09906 21441907 PMC3088773

[27] Huang da W, Sherman BT, Lempicki RA. Systematic and integrative analysis of large gene lists using DAVID bioinformatics resources. Nat Protoc. 2009;4(1):44–57. 10.1038/nprot.2008.211 . 19131956

[28] Wang J, Zhuang J, Iyer S, Lin X, Whitfield TW, Greven MC, et al Sequence features and chromatin structure around the genomic regions bound by 119 human transcription factors. Genome Res. 2012;22(9):1798–812. Epub 2012/09/08. 10.1101/gr.139105.112 22955990 PMC3431495

[29] Wu C, Orozco C, Boyer J, Leglise M, Goodale J, Batalov S, et al BioGPS: an extensible and customizable portal for querying and organizing gene annotation resources. Genome Biol. 2009;10(11):R130 10.1186/gb-2009-10-11-r130 19919682 PMC3091323

[30] Carriere L, Graziani S, Alibert O, Ghavi-Helm Y, Boussouar F, Humbertclaude H, et al Genomic binding of Pol III transcription machinery and relationship with TFIIS transcription factor distribution in mouse embryonic stem cells. Nucleic Acids Res. 2012;40(1):270–83. Epub 2011/09/14. 10.1093/nar/gkr737 21911356 PMC3245943

[31] Gaertner B, Zeitlinger J. RNA polymerase II pausing during development. Development. 2014;141(6):1179–83. 10.1242/dev.088492 24595285 PMC3943177

[32] Gilmour DS. Promoter proximal pausing on genes in metazoans. Chromosoma. 2009;118(1):1–10. 10.1007/s00412-008-0182-4 . 18830703

[33] Jonkers I, Kwak H, Lis JT. Genome-wide dynamics of Pol II elongation and its interplay with promoter proximal pausing, chromatin, and exons. Elife. 2014;3:e02407 10.7554/eLife.02407 24843027 PMC4001325

[34] Mavrich TN, Ioshikhes IP, Venters BJ, Jiang C, Tomsho LP, Qi J, et al A barrier nucleosome model for statistical positioning of nucleosomes throughout the yeast genome. Genome Res. 2008;18(7):1073–83. Epub 2008/06/14. 10.1101/gr.078261.108 18550805 PMC2493396

[35] Rhee HS, Pugh BF. ChIP-exo method for identifying genomic location of DNA-binding proteins with near-single-nucleotide accuracy. Curr Protoc Mol Biol. 2012;Chapter 21:Unit 21 4. Epub 2012/10/03. 10.1002/0471142727.mb2124s100 . 23026909 PMC3813302

[36] Li H, Durbin R. Fast and accurate short read alignment with Burrows-Wheeler transform. Bioinformatics. 2009;25(14):1754–60. Epub 2009/05/20. 10.1093/bioinformatics/btp324 19451168 PMC2705234

[37] Albert I, Wachi S, Jiang C, Pugh BF. GeneTrack—a genomic data processing and visualization framework. Bioinformatics. 2008;24(10):1305–6. Epub 2008/04/05. 10.1093/bioinformatics/btn119 . 18388141 PMC7058423

[38] Dunham I, Kundaje A, Aldred SF, Collins PJ, Davis CA, Doyle F, et al An integrated encyclopedia of DNA elements in the human genome. Nature. 2012;489(7414):57–74. Epub 2012/09/08. 10.1038/nature11247 22955616 PMC3439153

[39] Berger MF, Levin JZ, Vijayendran K, Sivachenko A, Adiconis X, Maguire J, et al Integrative analysis of the melanoma transcriptome. Genome Res. 2010;20(4):413–27. Epub 2010/02/25. 10.1101/gr.103697.109 20179022 PMC2847744

