# Supplementary figures and images for: Genomic Organization of Human Transcription Initiation Complexes
Source: PLoS One. 2016 Feb 11;11(2):e0149339. doi: 10.1371/journal.pone.0149339 (PMC4750860; doi:10.1371/journal.pone.0149339)

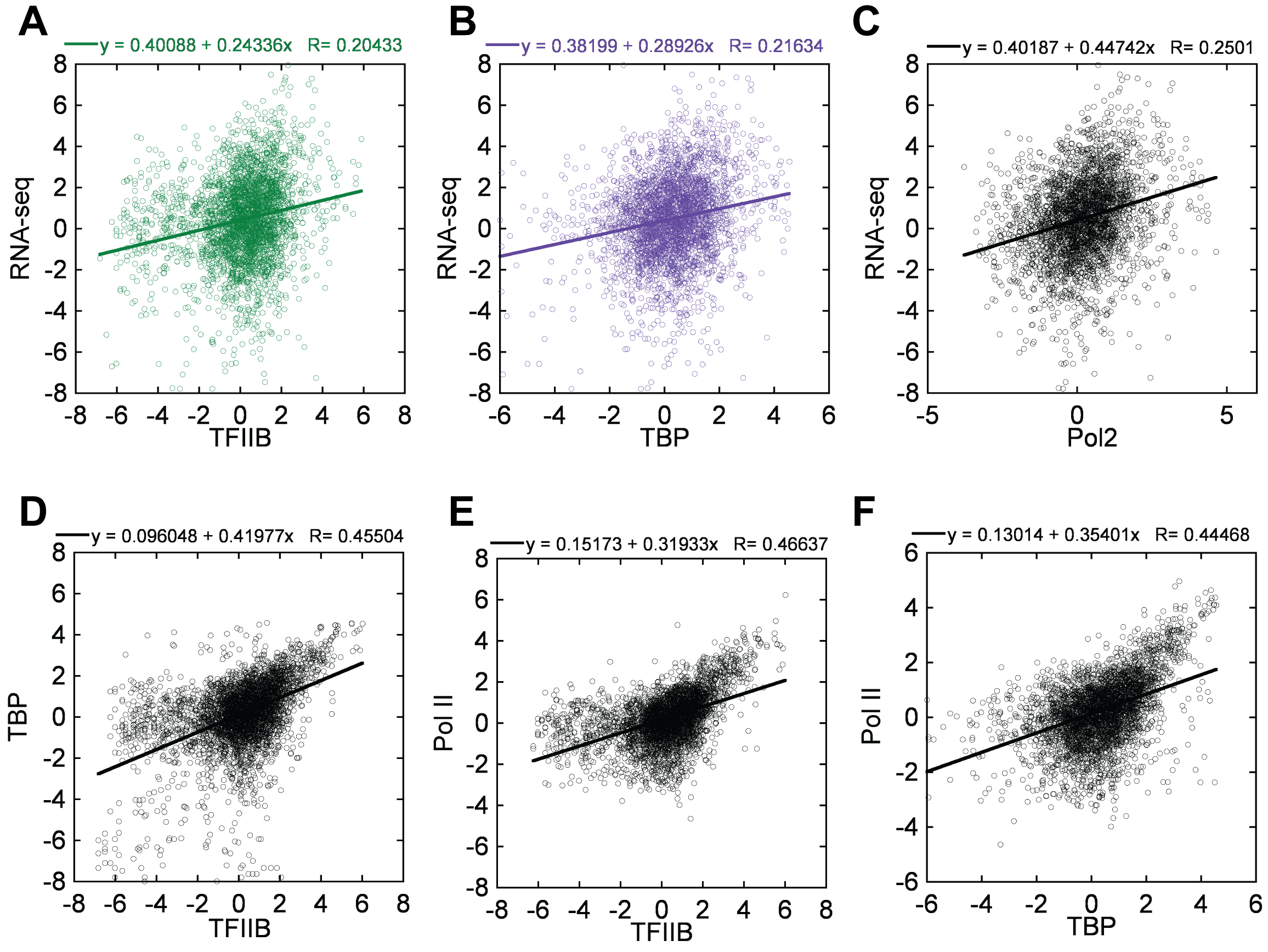

Supplement: S1 Fig — (A-C) Scatter plot and Pearson correlation fit of mRNA levels (RNA-seq) versus TFIIB/TBP/Pol II ChIP-exo occupancy levels, respectively, on a median-centered log2 scale. (D-F) Scatter plot and Pearson correlation fit of TFIIB/TBP/Pol II ChIP-exo occupancy levels versus each other on a median-centered log2 scale. (TIF) [file pone.0149339.s001.tif]

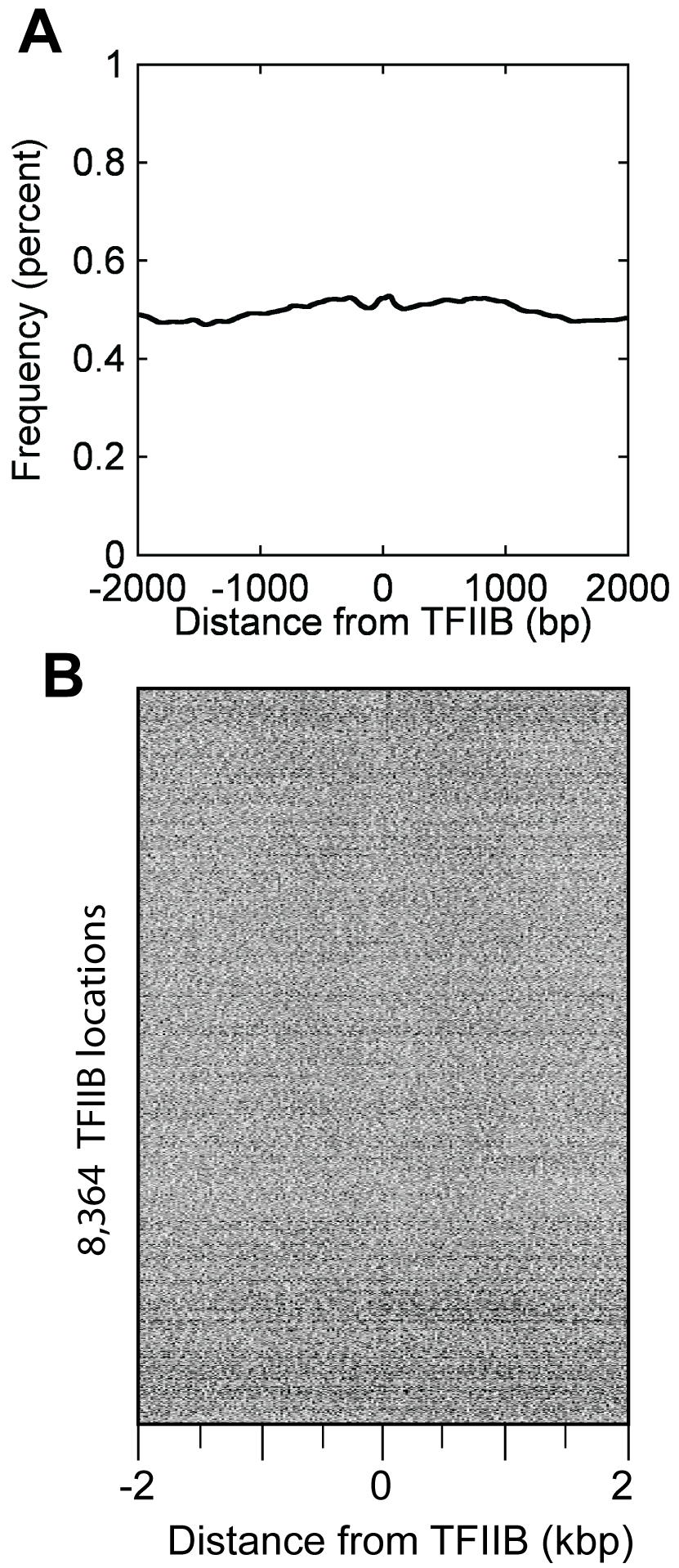

Supplement: S2 Fig — Input sequence tags are plotted relative to the 8,364 promoter-bound TFIIB locations from main Fig 2B. as an averaged composite distribution (A) or as a density plot (B). (TIF) [file pone.0149339.s002.tif]
